# Gonadal function in patients treated for Hodgkin′s disease in childhood

**DOI:** 10.2478/v10019-010-0034-8

**Published:** 2010-09-09

**Authors:** Lorna Zadravec Zaletel, Nevenka Bratanic, Berta Jereb

**Affiliations:** 1 Institute of Oncology, Department of Radiation Oncology, Ljubljana, Slovenia; 2 University Children Hospital Medical Center, Ljubljana, Slovenia

**Keywords:** gonadal function, late effects, childhood cancer, Hodgkin’s disease

## Abstract

**Background:**

The long-term survival of patients treated for Hodgkin`s disease (HD) in childhood is high and the chief concern is now being directed toward the late effects of the treatment, including the endocrine dysfunction.

**Patients and methods.:**

Testicular and ovarian functions were assessed in 64 long term survivors (24 females, 40 males) treated for HD in childhood in Slovenia between 1972 and 1994. At diagnosis they were 3–16 years old and had gonadal evaluation 4–27 years later at the age of 13–34. Fifty-four (84%) patients received chemotherapy (ChT), 49 in combination with radiation therapy (RT), 10 received RT alone. Gonadal function was assessed by the clinical examination and measurement of serum concentrations of estradiol and testosterone. Serum levels of LH and FSH were determined in the basal state and after the stimulation.

**Results:**

Primary hypogonadism (PH) was found in 30 (47%) patients. Twenty-four of 40 (60%) males had PH with evidence of damage of germinal epithelium, 4 of them had evidence of damage of Leydig cells (LC) and 10 had evidence of dysfunction of LC as well. PH was found in 6 of 24 (25%) females.

**Conclusions:**

After therapy for HD PH was more frequent in males than in females. Not only RT but also alkylating agents and procarbazine alone caused damage of LC. Age of patient at the time of treatment was not an important risk factor for gonadal toxicity. Pelvic RT in combination with ChT is the most important risk factor of the development PH both, in males and females.

## Introduction

The long-term survival of patients after the treatment of childhood cancer, especially Hodgkin`s diesease (HD), has greatly improved in the last few decades due to the effective treatment, especially multiagent chemotherapy (ChT).^1^ The chief concern is now being directed toward the late effects of the treatment^2^, which influence on patient’s quality of life and become more and more important in cancer treatment.^3^,^4^ Endocrine glands, gonads in particular, are very susceptible to damaging effects of anticancer therapy.^5^ The damaging effect of both ChT and radiotherapy (RT) on gonads is well known.^6^ In a study of 2283 long-term survivors of childhood cancer Byrne and colleagues found that RT below the diaphragm depressed fertility in both sexes for about 25%, ChT with alkylating agents with or without RT below the diaphragm depressed fertility by 60% in men, but in women alkylating agents therapy administered alone had no apparent effect on fertility.^7^

The aim of this study was to define the influence of cancer treatment on the gonadal status of 64 young adults treated for HD during childhood and adolescence in Slovenia.

## Material and methods

### Patients

Between 1972 and 1994, 104 patients were treated for HD during childhood (0–16 years of age) in Slovenia. Twenty-four patients had died, 4 were lost to follow-up. Seventy-six ptatients are regularly followed at the outpatient Clinic for Late Effects at the Institute of Oncology, Ljubljana. Twelve patients refused endocrinological evaluation, so 64 patients (24 females, 40 males) were included in our analysis. They were treated for HD at the age of 3–16 (median 13) years and had endocrinological evaluation 4–27 (median 10) years after the end of the treatment at the age of 13–34 (median 21) years. All patients were pubertal or postpubertal when studied (only two patients were younger than 16 years).

At diagnosis, 13 patients were in stage I (12 above the diaphragm), 23 in stage II (21 above the diaphragm), 25 in stage III and 3 in stage IV (2 involvement of the lung, one of the liver). Seven patients (5 boys, 2 girls) suffered from relapse. Forty-nine patients were treated with ChT and RT, 10 with RT and 5 with ChT as the only treatment modality. Fifty-four patients had combination ChT with MOPP [mechlorethamine, vincristine, procarbazine, prednisone], MOPP-ABV [mechlorethamine, vincristine, procarbazine, prednisone-doxorubicin, bleomycin, vinblastine] hybrid, MOPP/ABVD [mechlorethamine, vincristine, procarbazine, prednisone/doxorubicin, bleomycin, vinblastine, dacarbazine], LOPP [chlorambucil, vincristine, procarbazine, prednisone], COPP(A) [cyclophosphamide, vincristine, procarbazine, prednisone, (doxorubicin)] and OPPA [vincristine, procarbazine, prednisone, doxorubicin]^8^–^11^ (Table 1). Of the 59 patients treated with RT, 27 (19 boys, 8 girls) had RT above the diaphragm with 20–40 (median 30) Gy, 17 (8 boys and 9 girls) RT to the upper abdomen with 24–49 (median 30) Gy and 15 (11 boys, 4 girls) RT to the pelvis with 22–45 (median 30) Gy.

Twenty-eight (12 girls and 16 boys) patients had also staging laparotomy with splenectomy.

### Assessment of gonadal function

The patient’s data, regarding both, the diagnosis and the treatment, were collected from medical files, information concerning quality of life including attained educational level, marital status, employment and social life, past and present menstrual histories, the course of puberty and fertility histories were ascertained by the interview. The general physical examination was performed, height, weight and clinical abnormalities as well as Tanner stages of pubic hair and genital development were recorded. Each patient’s blood samples were analysed for basal concentrations of total testosterone (RIA, IMUNOTECH), estradiol (DELFIA-LKB) and prolactin (DELFIA-LKB). Concentrations of luteinizing hormone (LH) (DELFIA-LKB) and follicle stimulating hormone (FSH) (DELFIA-LKB) were determined before and 10, 20, 30, 60 minutes after *i.v.* administration of gonadotropin releasing hormone (50 mcg/m^2^) (LH-RH). Semen analyses were performed in 6 men.

Primary hypogonadism (PH) was defined as basal serum FSH and/or LH level above the normal upper limit and exaggerated response after the stimulation with LH-RH. In men, elevated basal serum FSH levels indicated germinal epithelium damage (GE-DA), while elevated LH levels (with/without reduced total testosterone levels) indicated Leydig cells (LC) damage (LC-DA). Normal basal values of LH and/or FSH and the exaggerated response after LH-RH stimulation were considered as a subclinical impairment of the gonadal function (SIG). The exaggerated response of FSH after LH-RH was considered as a dysfunction of germinal epithelium (GE-dys), while the exaggerated response of LH after LH-RH were considered as a dysfunction of LC (LC-dys). Low serum basal FSH and LH levels with the poor response after *i.v.* bolus of LH-RH was considered as secondary hypogonadism (SH).

## Results

### Males

We found PH in 24 of 40 males (60%); in 20 of 35 males (57%) who had primary treatment and in 4 of 5 males (80%) treated for the relapse (Table 2). All 24 males had the evidence of GE-DA, four of them had the evidence of LC-DA (low level of total testosterone in one) and ten had the evidence of LC-dys as well. Twenty-two of 24 males with PH had received combination ChT (all but one ≥ 6 cycles of AA and procarbazine (P) containing ChT) and RT (to the pelvis in 8), 2 had had pelvic RT only (Table 2). Patients with LC-DA or LC-dys had received somewhat higher cumulative doses of P (med. 7.4 g/m^2^) than those who had a normal LC function (med. 6.5 g/m^2^), while cumulative doses of alkylating agents, proportion of patients having received pelvic RT and ages at diagnosis did not differ among the two groups. Semen analyses were performed in 6 of 24 (25%) males with PH and all were azoospermic. Five males of the 24 with PH have children.

Three patients had SIG: one had isolated GE-dys, the second LC-dys and the third GE-dys in combination with LC-dys, all after the treatment with combined ChT (MOPP/ABVD x 6, MOPP x 4 and LOPP x 6) without pelvic RT. One of them has children. One patient had evidence of SH. He had been treated with RT to the neck and mediastinum (40 Gy) (Table 2).

Of 11 males who had had pelvic RT (9 to the iliacoinguinal region, 2 to the iliacal region), 9 in combination with ChT, 10 had PH. ChT alone was less gonadotoxic, causing PH in 14 of 29 (48%) males than ChT in combination with pelvic RT (PH in 8 of 9 (89%) males). Only one of these patients, who had received RT to the iliacal region only in combination with two cycles of MOPP, had normal gonadal function (Tables 2, 3).

The endocrinological evaluation was normal in 12 males. Seven of them had received ChT and RT, four RT only and one ChT only (Table 3).

Among 7 males having received 6 cycles of MOPP without pelvic RT only one had normal endocrine tests. He had received 6 cycles of ABVD as well. Among 10 males having received 6 cycles of LOPP without pelvic RT 4 had normal gonadal function.

Three patients had received 1 or 2 cycles of MOPP or OPPA and had normal testicular function (Table 2).

Age at diagnosis, follow-up time and age at endocrine evaluation were similar in the group of patients with PH and in the group of patients without endocrinological deficiences. The only difference between the groups was the treatment modality (Tables 2, 3).

### Females

We found PH in 6 of 24 (25%) females (Table 4), 3 of them had low levels of estrogen as well. All 6 had been treated with ChT and RT. Two of them had been treated with ChT (COPPA×6 and LOPP×6) and unilateral pelvic RT (24 and 30 Gy), one has primary amenorrhea, the other has irregular menstrual periods and one child. Four females with PH had received ChT (two 6 cycles of MOPP, one 6 cycles of LOPP, one ChT following protocol BFM 90 and 3 cycles of (C)OPP(A)) and nonpelvic RT, 3 upper abdominal. Two of these 4 patients have regular menstrual periods and children, one has irregular menses and one is in early menopause after having given birth to 2 children.

We found SIG in one female treated with 4 cycles of MOPP and neck RT. She has irregular menstrual periods and gave birth to one child (Table 4).

Seventeen females had normal gonadal function with regular menstrual periods; 14 of them had had ChT, 13 in combination with RT (2 pelvic), 3 had had nonpelvic RT only (Table 4, 5).

Among 6 females having received 6 or more cycles of MOPP ChT without pelvic RT, 4 had normal gonadal function (one even after 8 cycles of MOPP, 3 after ChT containing 6 cycles of ABVD as well). One female had normal gonadal function after 3 cycles of MOPP plus 3 cycles of LOPP. Of 2 females having received 6 cycles of LOPP without pelvic RT one had normal gonadal function (Table 5).

All of 5 females having received 6 cycles of MOPP/ABV hybrid regimen (in combination with unilateral pelvic RT in 2) had normal gonadal function (Table 5).

The group of females with PH and the group with a normal gonadal function did not differ regarding age at diagnosis, follow-up time or age at endocrine evaluation; they differ only by the mode of the treatment. Females with PH had received slightly larger cumulative doses of P (med. 7 g/m^2^) than those with a normal gonadal function (med. 5.5 g/m^2^). Females with PH had had abdominal RT in higher proportion (5 of 6 (83%) (pelvic RT in 2 of 6 (40%)) than females with normal gonadal function (abdominal RT in 8 of 17 (47%) (pelvic in 2 of 17 (12%)). ChT in combination with pelvic RT was more gonadotoxic, causing PH in 2 of 4 (50%) females, than ChT alone (PH in 4 of 20 (20%) females).

None of female patients had evidence of SH.

## Discussion

Our study is a population based study. As to our knowledge there is no international population based study of gonadal dysfunction after the treatment of HD in childhood. The study populations of most studies, dealing with this topic, are selected according to the type of treatment, age, gender or institution.

Several studies showed that in men basal FSH levels and FSH response to LH-RH correlated well with the sperm production.^12^–^16^ An increased FSH response to LH-RH can be the first manifestation of testicular damage^15^, although normal FSH levels do not rule out the possibility of azoospermia.^14^,^17^,^18^ In our study semen analyses in 6 patients with a high basal FSH level showed azoospermia.

Our findings are in concordance with data from other studies establishing that MOPP or MOPP-like combinations, such as MVPP (mechlorethamine,vinblastine, procarbazine and prednisone) and COPP induce azoospermia in 90–100% of patients with a 10–20% chance of recovery even 10 years after treatment.^17^,^19^–^24^ In our study 1 of 7 males had a normal gonadal function after receiving 6 cycles of MOPP. The recovery of spermatogenesis following MOPP therapy appears to be dose-related with 3 courses of MOPP representing a limiting gonadal exposure for the recovery, suggesting only a partial killing of germinal stem cells.^25^ Indeed, in our study we found a normal gonadal function in two males after having received 1 and 2 cycles of MOPP ChT. We found ChT according to the protocol LOPP less damaging for testicular function than MOPP, causing GE-DA in 5 of 10 males having received 6 cycles, a finding not published elsewhere to our knowledge.

Four males had evidence of LC-DA, 12 had LC-dys. All but two of them had evidence of GE-DA as well. Only 1 of 4 males with LC-DA and 4 of 12 males with LC-dys had had pelvic RT, the others had received ChT with nonpelvic RT, indicating the adverse effect of chemotherapeutic agents on Leydig cells. This is supportive to the observations of Romerius and colleagues^26^ and Mustieles and colleagues.^12^ The possible explanation for a high incidence of the dysfunction of LC in males with damage of germinal epithelium (in 10 of 20) is coexistence of compensated LC failure with the germ cell depletion observed in males not treated for malignancies.^27^

Besides AA and P, pelvic RT turned out to be very gonadotoxic. Ten of eleven males, who had had pelvic RT, had PH. Two of those had had RT only, inverted Y, with doses of 40 Gy respectively, without special shielding of testes. It is well known that a cumulative dose of 200 cGy in multiple fractions may cause azoospermia and that is the dose of scattered radiation that the testes could receive from an inverted Y field.^28^

Five of 24 males with GE-DA fathered children indicating that they are not azoospermic but possibly oligospermic and fertile. Hoorweg-Nijman and colleagues found elevated levels of FSH compatible with normospermia.^16^ FSH levels may provide an estimate of possible impaired spermatogenesis, however only the semen analysis is confirmatory assessment of the male gonadal function.

We have no explanation for SH in male treated with RT above the diaphragm (not including hypophysis).

We found PH in 6 of 24 (25%) females. Only one of them is amenorrhoic (after 6 cycles of COPPA). There are data of adverse effects of ChT that is in use for HD, on ovarian function in adult females, but very little on ovarian function in girls. In the study of Ortin and colleagues 2 of 18 girls were amenorrhoic after having received 6 or more cycles of MOPP.^29^ In our study none of 6 girls is amenorrhoic after 6 or more cycles of MOPP, but 2 have evidence of the ovarian damage while retaining fertility.

Ionizing radiation is toxic to the ovaries. The dose of 20Gy is necessary for inducing ovarian failure in girls, but only one third of this dose will produce the same effect in women over 40 years.^30^ After the abdominal irradiation in childhood with doses of 20–30 Gy, the ovarian failure was found in 17 of 18 females.^31^ Four females in our series had had unilaterally pelvic RT. Half of them had evidence of PH, but all had received ChT with AA and P as well. After RT of paraaortic lymph nodes the estimated ovarian dose is about 6 % of the prescribed dose (in the range of 100 cGy) and this dose of radiation can cause transient disturbances of menstrual cycle. Haie-Mader and colleagues analyzed the ovarian function in 134 females who had ovarian transposition during the treatment for HD or gynecological cancer and showed that the age over 25 years, MOPP ChT and total dose to the ovaries higher than 5 Gy are important risk factors for the ovarian castration.^32^

Four females in our study had elevated basal and peak FSH levels while retaining menstrual periods. Four of 6 with PH even gave birth to children. Sherman and Korenman stated that increased FSH concentration with regular menstrual cycles might be consistent with the deficient production of inhibin or an inhibin-like hormone by the partially damaged ovary.^33^ It might announce the risk of premature menopause in these females, which occurred in one of our patients. Byrne and colleagues found out that the treatment for cancer during adolescence carries a substantial risk of early menopause.^34^ In a study of Sklar and colleagues risk factors for nonsurgical premature menopause in survivors of childhood cancer were attained age, exposure to increasing doses of radiation to the ovaries, increasing alkylating agents score and a diagnosis of HD.^35^

It seems that in males with HD therapy is not the only cause of hypogonadism. Vigersky and colleagues found a low sperm count or sperm motility in about one-third of male patients with HD before starting ChT.^36^ Rueffer and colleagues found in their study semen abnormalities in 70% of patients before the onset of the treatment for HD.^37^ Unlike in males it seems that there is no adverse effect of Hodgkin’s disease on the female gonadal function. In a study of Chapman and colleagues, namely, histories and pretreatment ovarian biopsy specimens indicated the normal fertility before the therapy for HD.^21^

## Conclusions

After the therapy for HD hypogonadism is more frequent in males than in females.

Six or more cycles of MOPP causes PH in more than half patients. This kind of treatment is more toxic for males than for females. Six cycles of LOPP seems less gonadotoxic than 6 cycles of MOPP. MOPP/ABV hybrid regimen is not gonadotoxic for females.

Not only RT but also alkylating agents and procarbazine alone cause damage of Leydig cells.

Elevated levels of FSH are compatible with the normal fertility in males and females.

The age of patient at the time of the treatment doesn’t emerge as an important risk factor for gonadal toxicity, caused by therapy.

Pelvic RT in combination with ChT is the most important damaging factor causing the gonadal dysfunction both, in males and females.

## Figures and Tables

**TABLE 1:** Chemotherapy in 54 patients treated for Hodgkin’s disease

	**N° of patients**				
	**Alone**	**In combination with**			**Total**
		**LOPP**	**ABV(D)**	**COPP(A)**	
**MOPP**	11	2	10 ♣	1	24
**LOPP**	14		1	1	16
**MOPP/ABV hybrid**	8				8
**COPP(A)**	3				3
**OPPA**	1			2 ♥	3
**Total N° of patients**	37	2	11	4	54

♣ 4 patients received also LOPP, 1 patient COPPA

♥ 1 female, missdiagnosed as having non-Hodkin’s lymphoma, received chemotherapy following protocol BFM 90 as first treatment

LOPP = chlorambucil, vincristine, procarbazine, prednison; ABV(D) = doxorubicin, bleomycin, vinblastine, (dacarbazine); COPP(A) = cyclophosphamide, vincristine, procarbazine, prednisone, (doxorubicin); MOPP = mechlorethamine, vincristine, procarbazine, prednisone; OPPA = vincristine, procarbazine, prednison, doxorubicin

**TABLE 2:** Gonadal function according the type of treatment in 40 males

**Type of treatment**	**N° of patients**				
	**Gonadal function**				**Total**
	**PH**	**SIG**	**SH**	**Normal**	
**Pelvic RT + ChT**	8			1♦	9
**Pelvic RT alone**	2			0	2
≥ **6 c (AAP) ChT with nonpelvic RT**	13	1		4	18
≥ **6 c (AAP) ChT, no RT**		1		1	2
≤ **5 c (AAP) ChT with nonpelvic RT**	1♣	1		2	4
**Nonpelvic RT alone**			1	4	5
**Total N° of patients**	24	3	1	12	40

♣ pt received 3 cycles (c) of LOPP

♦ RT to the iliacal region only

PH = primary hypogonadism; SIG = subclinical impairment of gonadal function; SH = secondary hypogonadism; RT = radiotherapy; ChT = chemotherapy; AAP = ChT, containing alkylating agents and procarbazine (P) (MOPP, MOPP-ABV hybrid, MOPP/ABVD, LOPP, COPP(A) and OPPA)

**TABLE 3:** Therapy for Hodgkin’s disease in twelve males with normal endocrine tests

**Type of treatment**		**No of patients with normal gonadal function**	**Total N° of patients with the same type of treatment**
**ChT**	**RT**		
**MOPP/ABVD ×6**	none/nonpelvic	1	7 ♣ ♥
**LOPP × 6**	nonpelvic	4	10 ♣
**MOPP × 2**	iliacal region (30 Gy)	1	1
**MOPP × 1**	nonpelvic	1	1
**NONE**	nonpelvic	4	5 ♠
**OPPA × 2**	nonpelvic	1	1
**TOTAL**		12	25

♥ also patients receiving 6 cycles of MOPP only

♣ 1 patient had subclinical impairment of gonadal function

♠ 1 patient had secondary hypogonadism

ChT = chemotherapy; RT = radiotherapy; MOPP = mechlorethamine, vincristine, procarbazine, prednisone; ABVD = doxorubicin, bleomycin, vinblastine, dacarbazine; LOPP = chlorambucil, vincristine, procarbazine, prednison; OPPA = vincristine, procarbazine, prednisone, doxorubicin

**TABLE 4:** Gonadal function according the type of treatment in 24 females

**Type of treatment**	**N° of patients**			
	**Gonadal function**			**Total**
	**PH**	**SIG**	**Normal**	
**Pelvic RT +** ≥ **4 c (AA) ChT**	2		2	4
≥ **6 c (AA) ChT with nonpelvic RT**	4	0	7	11
≥ **6 c (AA) ChT, no RT**	0	0	3	3
≤ **5 c (AA) ChT with nonpelvic RT**	0	1	2	3
**Nonpelvic RT alone**			3	3
**Total N° of patients**	6	1	17	24

PH = primary hypogonadism; SIG = subclinical impairment of gonadal function; RT = radiotherapy; ChT = chemotherapy; AA = ChT, containing alkylating agents (MOPP, MOPP-ABV hybrid, MOPP/ABVD, LOPP, COPP(A) and OPPA)

**TABLE 5:** Therapy in 17 females with normal endocrine tests

**Type of treatment**		**N° of patients with normal gonadal function**	**Total N° of patients with the same type of treatment**
**ChT**	**RT**		
**MOPP × 8**	nonpelvic	1	3 ♣
**MOPP/ABV hybrid × 6**	nonpelvic	3	3
**MOPP/ABV hybrid × 6**	pelvic-unil.(24Gy)	1	1
**MOPP/ABVD ×6**	none	2	2
**MOPP/ABV×6+ABVD×6+LOPP×6**	pelvic-unil.(22Gy)	1	1
**MOPP/ABVD × 6 + LOPP × 3**	none	1	1
**MOPP × 3 + LOPP × 3**	nonpelvic	1	1
**LOPP × 3 +ABV × 2**	nonpelvic	1	1
**COPPA × 6**	nonpelvic	1	1
**LOPP × 6**	nonpelvic	1	2
**COPP × 2 + OPPA × 2**	nonpelvic	1	1
**None**	nonpelvic	3	3
**Total**		17	20

♣ 2 patients with primary hypogonadism received 6 cycles of MOPP

ChT = chemotherapy; RT = radiotherapy; MOPP = mechlorethamine, vincristine, procarbazine, prednisone; ABVD = doxorubicin, bleomycin, vinblastine, dacarbazine; LOPP = chlorambucil, vincristine, procarbazine, prednison; COPP = cyclophosphamide, vincristine, procarbazine, prednisone; OPPA = vincristine, procarbazine, prednisone, doxorubicin;
